# Analysis of Microbial Diversity in Soil under Ginger Cultivation

**DOI:** 10.1155/2017/8256865

**Published:** 2017-10-23

**Authors:** Yiqing Liu, Lin Wu, Xingwen Wu, Honghai Li, Qinhong Liao, Xiaojing Zhang, Zhiqiang Sun, Wenhua Li

**Affiliations:** ^1^Chongqing Key Laboratory of Economic Plant Biotechnology, Collaborative Innovation Center of Special Plant Industry in Chongqing, Institute of Special Plants, College of Forestry & Life Science, Chongqing University of Arts and Sciences, Yongchuan, Chongqing 402160, China; ^2^Liangping Agro-Technical Extension Station, Liangping, Chongqing 405200, China; ^3^Chongqing Engineering and Technology Research Center for Utilization of Ginger Resource, Chongqing Tianpei Agro-Tech Co. Ltd., Yongchuan, Chongqing 402189, China; ^4^Yantai Lvyun Biotechnology Co. Ltd., Yantai 264003, China

## Abstract

Ginger is a perennial monocotyledonous herb, which can be used as both a vegetable and a medicinal plant. However, it is susceptible to various plant pathogens. Microbial diversity in soil is related closely to the health and productivity of plant crops including ginger. In the current study, we compared microbial diversity from soil samples under ginger cultivation (disease incidence of >50% [relatively unhealthy sample] versus disease incidence of <10% [relatively healthy sample]). The bacterial and fungal taxa were analyzed by Illumina-based sequencing, with 16S and ITS identification, respectively. Both bacterial and fungal OTUs were significantly more in the healthy soil sample than the unhealthy sample. Moreover, the dominant bacterial and fungal genera were detected to be different in each sample.* Rhodanobacter* and* Kaistobacter* were the dominant bacterial genera in the healthy sample, while* Rhodoplanes *and* Bradyrhizobium* were the dominant genera in the unhealthy sample. For fungal analysis,* Cladosporium*,* Cryptococcus*, and* Tetracladium* were the dominant genera in the healthy sample, while* Lecanicillium*,* Pochonia*, and* Rhodotorula* were the dominant genera in the unhealthy sample. Collectively, the basic information of microbial diversity in ginger soil is helpful for elucidating the ginger-microbe interactions and potentially selecting suitable plant growth-promoting rhizobacteria and biocontrol agents for ginger production.

## 1. Introduction

Ginger* (Zingiber officinale)* is a perennial monocotyledonous herb, which can be used as both a vegetable and a medicinal plant. It is widely employed in Chinese, Ayurvedic medicines and home remedies since antiquity for many ailments including pain, inflammation, and gastrointestinal disorders [1]. However, ginger is subject to various diseases during growth stages [2–5]. The occurrence of these diseases results in significant yield reductions in ginger. For instance,* Enterobacter cloacae* causes ginger rhizome rot [6], while* Erwinia chrysanthemi* is the causal agent of ginger soft rot [7]. Root-rot disease, caused by the fungal pathogen,* Pythium myriotylum*, was reported to decimate ginger in field plantings, when temperatures ranged from 26 to 30°C and the soil was saturated with water due to continuous rainfall [8]. Ginger rhizomes infected with* Fusarium oxysporum* exhibit yellow shoots and die after a few weeks [9], and it is also a devastating postharvest disease for stored ginger [10].

Soil microbial community plays an important role in nutrient mobilisation and uptake for plant. They promote plant growth and suppress disease by their various activities, like phosphate and sulphate solubilisation, plant growth promotion, siderophore production, nitrogen fixation, denitrification, immune modulation, signal transduction, and pathogen control [11]. The objectives of the present study were to analyze and compare the microbial diversity from the soil under the cultivation of ginger with low disease incidence (healthy sample) and high disease incidence (unhealthy sample). Specially, total microbial DNA of ginger soil samples was purified and analyzed by Illumina-based sequencing. The bacterial and fungal communities were further compared and the vertically transmitted bacterial and fungal taxa were elucidated in the study.

## 2. Material and Methods

### 2.1. Soil Sampling and DNA Extraction

The soil samples were collected from the organic ginger field in Yongchuan, Chongqing, China (N29°10′57.80′′, E105°50′1.77′′), in Sep, 2016. This organic farm with total of 50,000 m^2^ area is divided into 100 planting units with each of 500 m^2^ area. When the gingers were harvested in Sep, 2016, it was found that the disease incidence varied among each planting unit. In order to analyze the microbial diversity, the rhizospheric soil samples were collected from two groups (healthy group versus disease group). Soil samples were collected at a depth of approximately 15 cm, in sterile polythene bags, and stored in refrigerator until DNA extraction. In Group I, the soil samples were taken from nine ginger-planting units in which the disease incidence was lower than 10%; in Group II, the soil samples were taken from nine ginger-planting units in which the disease incidence was more than 50%.

The soil samples from the two groups above were used for total DNA extraction. The total DNA was extracted using EZNA® Soil DNA Kit (Omega Bio-Tek, USA) according to the manufacture's instruction. Briefly, about 1 g soil sample was added to 15 mL centrifuge tube with glass beads. Total DNA was obtained after the procedures of lysis, centrifugation, binding on DNA binding column, elution, and purification. Total DNA concentration and purity were monitored on 1% agarose gels.

### 2.2. Amplicon Generation and Illumina MiSeq Sequencing

The pair of primers 515F (5′-GTGCCAGCMGCCGCGGTAA-3′) and 907R (5′-CCGTCAATTCCTTTGAGTTT-3′) were used to amplify the V4-V5 regions, a hypervariable area of the 16S rRNA gene. The primers ITS5-1737F (5′-GGAAGTAAAAGTCGTAACAAGG-3′) and ITS2-2043R (5′-GCTGCGTTCTTCATCGATGC-3′) targeting the ITS1 regions of fungal rRNA genes were adopted to analyze fungal taxa [12]. Both forward and reverse primers were tagged with adapter, pad, and linker sequencing. Each barcode sequence was added to the reverse primer for pooling multiple samples into one run of sequencing. All PCR amplifications were performed in terms of previously reported method [13]. The reaction conditions were as follows: an initial denaturation at 98°C for 1 min, each of 30 cycles at 98°C for 10 s, 55°C for 30 s, and 72°C for 60 s, with a final extension at 72°C for 5 min. After mixing the PCR products of the triplicate, detection was implemented by 2% (w/v) agarose gel electrophoresis, and then the PCR products were purified by the AxyPrep Gel Extraction Kit (Axygen, USA). Amplicons from each reaction mixture were quantified fluorometrically, normalized, and pooled at equimolar ratios based on the concentration of each amplicon. The sequencing libraries were generated using NEB Next Ultra™ DNA Library Prep Kit for Illumina (New England Biolabs) following manufacturer's recommendations, and index codes were added. The library quality was assessed on the Qubit^@^ 2.0 Fluorometer (Thermo Scientific) and Agilent Bioanalyzer 2100 system (Agilent Technologies). Finally, the libraries were sequenced on an Illumina MiSeq platform [14, 15].

### 2.3. Data Preprocessing

All sequence reads with the same tag were assigned to the same sample according to the unique barcodes (raw tags). The raw tags were further filtered by clean tags, and the quality of clean tags was detected by Qiime (http://qiime.org/index.html) [16, 17]. Rarefaction analysis was implemented based on MOTHUR package, using operational taxonomic units (OTUs) grouped at 97% sequences similarity [18]. The numbers of randomly selected sequences and corresponding OTUs under the sobs diversity index were employed as the variables.

## 3. Results and Discussion

The soil provides a great variety of microhabitats for myriad organisms of different size, physiological activity, behavior, and ecosystem function [19]. The extent of the diversity of microorganisms in soil is critical to the maintenance of health and quality of soil and plant, as a wide range of microorganisms is involved in important soil functions [20]. Recently, there have been a few studies on reports of ginger diseases [14, 15, 21, 22]. However, to the best of our knowledge, the reports on soil microbes in ginger fields are limited. In the present study, we compared the microbial diversity from soil samples of ginger-planting field (ginger with low disease incidence [healthy sample] versus ginger with high disease incidence [unhealthy sample]). The data of this present study showed that both bacterial and fungal OTUs were significantly more in the healthy soil sample than the unhealthy sample (Table 1). These findings were consistent with the previous study about microbial diversity in the soil under cultivation of potato [23] and maize [24].

**Figure 1:**
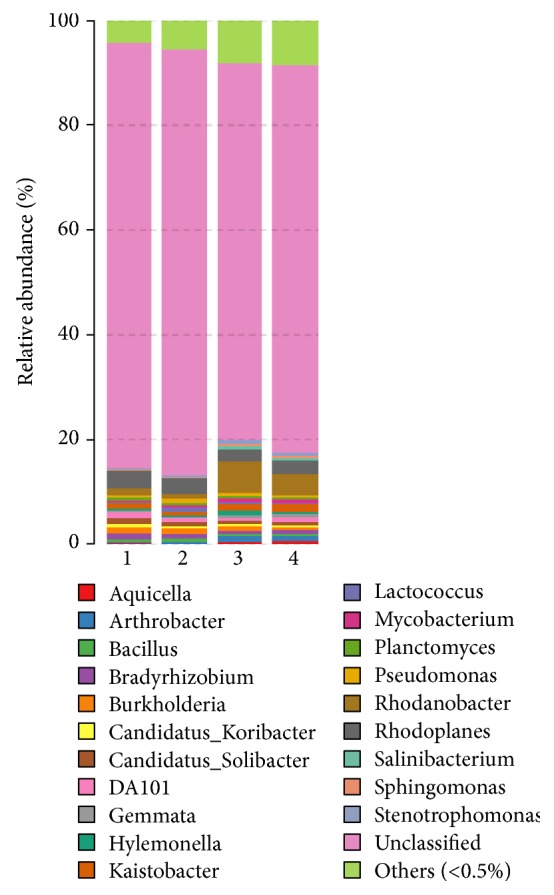
Relative abundance of bacterial genera. Samples 1 and 2 represent the two replicates of relatively unhealthy soil samples, while Samples 3 and 4 represent the two replicates of relatively healthy soil samples.

**Figure 2:**
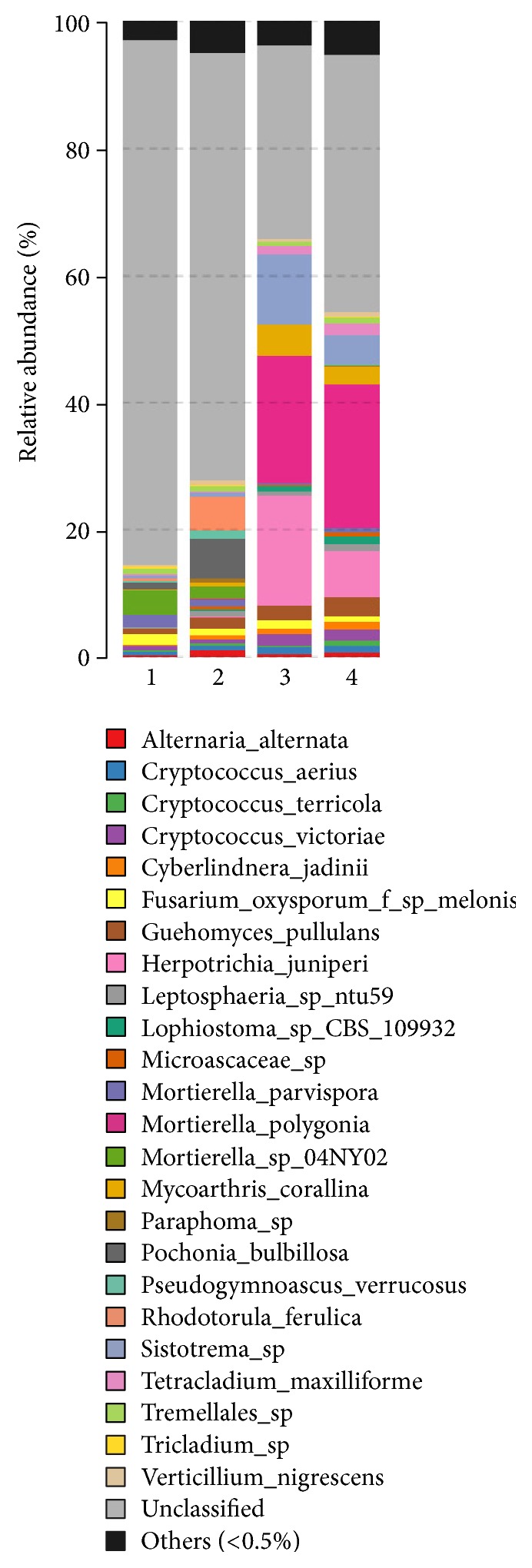
Relative abundance of fungal genera. Samples 1 and 2 represent the two replicates of relatively unhealthy soil samples, while Samples 3 and 4 represent the two replicates of relatively healthy soil samples.

More specially, the dominant bacterial and fungal genera were detected to be different in each sample.* Rhodanobacter* and* Kaistobacter* were the dominant bacterial genera in the healthy sample, while* Rhodoplanes *and* Bradyrhizobium* were the dominant genera in the unhealthy sample (Figure 1).* Rhodanobacter spathiphylli* sp. Nov. was isolated from a gamma proteobacterium isolated from the roots of* Spathiphyllum *plants and showed biocontrol activity towards the root-rot plant pathogen* Cylindrocladium spathiphylli *[25]. For fungal analysis,* Cryptococcus*,* Cladosporium*, and* Tetracladium* were the dominant genera in the healthy sample, while* Lecanicillium*,* Pochonia*, and* Rhodotorula* were the dominant genera in the unhealthy sample (Figure 2).* Cryptococcus *genus has been reported to be with potential plant growth-promoting traits [26], and* Lecanicillium fungicola *is a causal agent of dry bubble disease for many crops [27].

## 4. Conclusions

The current knowledge concerning the microbial diversity, particularly bacteria and fungi in ginger field, has the potential to understand the complex ecosystem of microbe-microbe and microbe-ginger interaction. The dominant bacterial and fungal genera in the ginger soil samples (healthy sample versus unhealthy sample) identified have the potential to explore biocontrol agents and pathogens. This study provides implications for maintenance of soil health and sustainable agriculture of ginger production.

## Figures and Tables

**Table 1:** Number of OTUs in the samples.

Microbial samples	OTUs
Bacteria	
Sample 1	1347
Sample 2	1179
Sample 3	1506
Sample 4	1661
Fungi	
Sample 1	183
Sample 2	196
Sample 3	202
Sample 4	221

*Note*. Samples 1 and 2 represent the two replicates of soil samples under ginger cultivation with disease incidence of >50% (relatively unhealthy sample), while Samples 3 and 4 represent the two replicates of soil samples under ginger cultivation with disease incidence of <10% (relatively healthy sample).
